# Impact of food processing and detoxification treatments on mycotoxin contamination

**DOI:** 10.1007/s12550-016-0257-7

**Published:** 2016-08-23

**Authors:** Petr Karlovsky, Michele Suman, Franz Berthiller, Johan De Meester, Gerhard Eisenbrand, Irène Perrin, Isabelle P. Oswald, Gerrit Speijers, Alessandro Chiodini, Tobias Recker, Pierre Dussort

**Affiliations:** 1 Molecular Phytopathology and Mycotoxin Research, Georg-August-University Göttingen, Grisebachstrasse6, 37077 Göttingen, Germany; 2 Barilla G. R. F.lli SpA, Advanced Laboratory Research, via Mantova 166, 43122 Parma, Italy; 3 Christian Doppler Laboratory for Mycotoxin Metabolism, Department IFA-Tulln, University of Natural Resources and Life Sciences, Vienna, Konrad-Lorenz-Straße 20, 3430 Tulln, Austria; 4 Cargill R&D Center Europe, Havenstraat 84, B-1800 Vilvoorde, Belgium; 5 Department of Chemistry, Division of Food Chemistry and Toxicology, Germany (retired), University of Kaiserslautern, P.O.Box 3049, 67653 Kaiserslautern, Germany; 6 Nestlé Research Center, Vers-chez-les-Blanc, PO Box 44, 1000 Lausanne 26, Switzerland; 7 INRA, UMR 1331 ToxAlim, Research Center in Food Toxicology, 180 chemin de Tournefeuille, BP93173, 31027 Toulouse, France; 8 Université de Toulouse, INP, UMR1331, Toxalim, Toulouse, France; 9 General Health Effects Toxicology Safety Food (GETS), Winterkoning 7, 34353 RN Nieuwegein, The Netherlands; 10 International Life Sciences Institute-ILSI Europe, Avenue E. Mounier 83, Box 6, 1200 Brussels, Belgium

**Keywords:** Mitigation, Natural toxins, Physical methods, Chemical treatment, Biological detoxification, Decontamination

## Abstract

Mycotoxins are fungal metabolites commonly occurring in food, which pose a health risk to the consumer. Maximum levels for major mycotoxins allowed in food have been established worldwide. Good agricultural practices, plant disease management, and adequate storage conditions limit mycotoxin levels in the food chain yet do not eliminate mycotoxins completely. Food processing can further reduce mycotoxin levels by physical removal and decontamination by chemical or enzymatic transformation of mycotoxins into less toxic products. Physical removal of mycotoxins is very efficient: manual sorting of grains, nuts, and fruits by farmers as well as automatic sorting by the industry significantly lowers the mean mycotoxin content. Further processing such as milling, steeping, and extrusion can also reduce mycotoxin content. Mycotoxins can be detoxified chemically by reacting with food components and technical aids; these reactions are facilitated by high temperature and alkaline or acidic conditions. Detoxification of mycotoxins can also be achieved enzymatically. Some enzymes able to transform mycotoxins naturally occur in food commodities or are produced during fermentation but more efficient detoxification can be achieved by deliberate introduction of purified enzymes. We recommend integrating evaluation of processing technologies for their impact on mycotoxins into risk management. Processing steps proven to mitigate mycotoxin contamination should be used whenever necessary. Development of detoxification technologies for high-risk commodities should be a priority for research. While physical techniques currently offer the most efficient post-harvest reduction of mycotoxin content in food, biotechnology possesses the largest potential for future developments.

## Introduction

Toxic secondary metabolites produced by fungi belong to the most toxic contaminants regularly occurring in a wide range of food commodities (Bennett and Klich 2003). Most countries responded to this threat by establishing and enforcing maximum levels for mycotoxins in food (European Commission 2006; van Egmond et al. 2007). Setting maximum levels is based on toxicity assessment and exposure data but it also takes supply and demand into account. Raw materials are usually tolerated to have higher contamination levels (except for products intended for direct human consumption) than finished products. The rationale behind this is a dilution effect when formulating with non-contaminated ingredients in preparation of the final product as well as of the potential mitigation effects due to processing. In both cases, the mycotoxin concentration in the finished product will be lower than in the raw material.

**Fig. 1:**
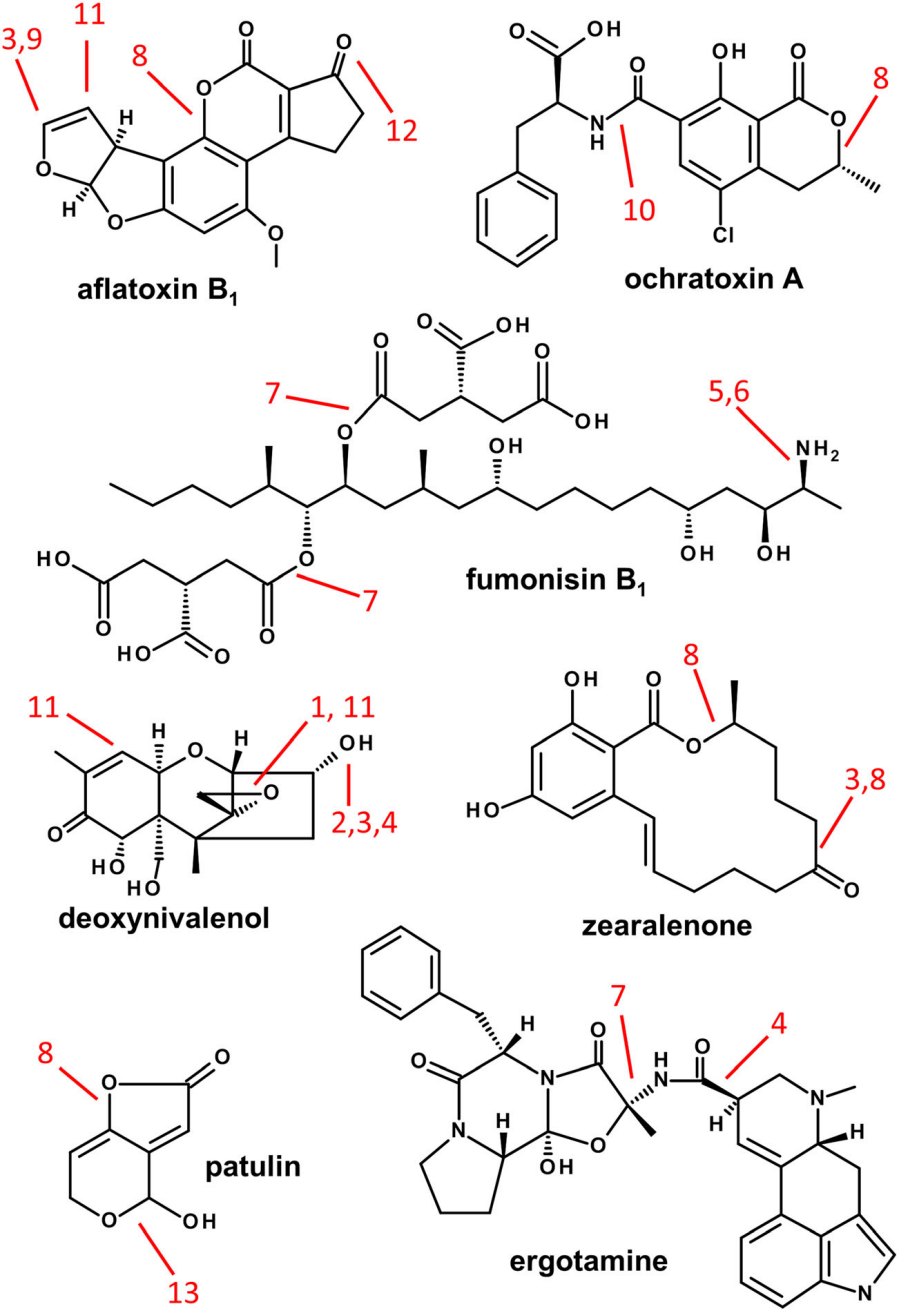
Chemical structures of major mycotoxins and modification due to food processing. *1* de-epoxidation, *2* acetylation, *3* oxidation, *4* epimerization, *5* deamination, *6* glucosylation, *7* hydrolysis, *8* lactone cleavage (hydrolysis), *9* hydroxylation, *10* peptide cleavage, *11* sulfonation, *12* reduction, *13* ether cleavage

Spoilage and toxin formation can occur already on the field and during storage of agricultural commodities or processed food. This article focuses on food, but results obtained on feed will be considered when they can be used to estimate the efficiency of mitigation strategies potentially useful for food. A variety of fungal species mostly from the genera *Aspergillus*, *Penicillium*, *Fusarium*, *Alternaria*, or *Claviceps* are known to produce mycotoxins. Most important in terms of toxicity and occurrence are aflatoxins B_1_, B_2_, G_1_, and G_2_ (AFB_1_, AFB_2_, AG_1_, AFG_2_); ochratoxin A (OTA); fumonisins B_1_, B_2_, and B_3_ (FB_1_, FB_2_, FB_3_); deoxynivalenol (DON) and other trichothecenes; zearalenone (ZEN); patulin (PAT); and ergot alkaloids (EAs), which are briefly characterized in Table 1, while their chemical structures are shown in Fig. 1.

**Table 1:** Major mycotoxins and their producers, affected crops, adverse health effects and guidance values

Mycotoxin	Major producing fungi	Main affected crops	Principal adverse effects	Health-based guidance value (HBGV)
Aflatoxins FB_1_, FB_2_, FG_1_, FG_2_; metabolite AFM_1_ in milk	*Aspergillus parasiticus*, *A. flavus* (JECFA 2001a)	Peanuts, nuts, maize, cottonseed, wheat, barley, cocoa beans, rice, copra, dried fruits, spices, figs, crude vegetable oils (IARC 2012; EFSA 2007; JECFA 1999)	Extremely potent toxins and genotoxic carcinogens (after metabolic converstion to 8,9-epoxides in the liver); classified as carcinogenic to humans, AFM_1_ as possibly carcinogenic to humans (EFSA 2007; IARC 2012; JECFA 1999, 2001a)	Because of carcinogencity, exposure should be kept as low as reasonably achievable. No official HBGV
Ochratoxin A (OTA)	*Aspergillus alutaceus*, *Aspergillus carbonarius*, *Penicillium verrucosum* (EFSA 2006)	Grain, legumes, oleaginous seeds, peanuts, cashews, dried fruits, coffee, wine, grape juice, cocoa, spices, meat products (JECFA 2001a; EFSA 2006)	Nephrotoxic, renal tumors in rodents at high doses (EFSA 2006, JECFA 2001a, IARC 1993); classified as carcinogenic in experimental animals and possibly humans (IARC 1993)	PTWI 120 ng/kg BW/day (EFSA 2006) and 100 ng/kg BW/day (JECFA 2001a)
Fumonisins B_1_, B_2_, and B_3_ (FB1, FB2, FB3)	*Fusarium verticillioides*, *F. proliferatum*, *Aspergillus niger* (EFSA 2005; JECFA 2001a, 2012)	Maize (*Fusarium* spp.), grapes (*A. niger*) (EFSA 2005; JECFA 2001a, 2012)	Inhibit sphingolipid biosynthesis; induction of apoptosis, tumors in rodents (EFSA 2005; JECFA 2001a; SCF 2003, IARC 2002), putative teratogenicity; FB_1_ classified as possibly carcinogenic to humans (IARC 2002)	Group PMTDI (JECFA 2001a, 2012) and group TDI (SCF 2003) 2 μg/kg BW/day for FB_1_, FB_2_, and FB_3_ alone or in combination
Deoxynivalenol (DON) and its acetylated derivates (3- and 15-acetyl-DON)	*F. graminearum*, *F. culmorum* (EFSA 2004, 2011a; JECFA 2001a, 2011)	Wheat, maize, barley, oats, rye; less often rice, sorghum and triticale (EFSA 2004, 2011a; JECFA 2001a, 2011)	Feed refusal, vomiting, and diarrhea; reduced growth; thymus, spleen, heart, liver, and immune system affected at higher doses (EFSA 2004; IARC 1993; JECFA 2001a; SCF 2002); not classifiable as to carcinogenicity to humans, (IARC 1993)	TDI 1 μg/kg BW/day for DON (SCF 2002, EFSA 2004); group PMTDI 1 μg/kg BW/day; ARfD 8 μg/kg BW/day for DON and its acetylated derivatives (JECFA 2011)
Other trichothecenes, e.g., T-2 toxin, HT-2 toxin, nivalenol (NIV)	*F. sporotrichioides*, *F. langsethiae* (JECFA 2001a), *F. poae* and *F. cerealis*, *F. culmorum* and *F. graminearum* (EFSA 2013)	Cereals (EFSA 2011a)	Acute effects of T-2 similar to high dose radiation (diarrhea, hemorrhage, hematotoxicity, and immune suppression) (JECFA 2001a, EFSA 2011a); toxicological profile of NIV similar (EFSA 2013); not classifiable as to carcinogenicity to humans (IARC 1993)	Group TDI 0.1 μg/kg BW/day (EFSA 2011a) and group PMTDI 0.06 μg/kg BW/day (JECFA 2001a) for T-2 and HT-2 toxins combined. TDI 1.2 μg/kg BW/day for NIV (EFSA 2013)
Zearalenone (ZEN)	*Fusarium* spp. (JECFA 2000, EFSA 2011b)	Worldwide in all types of grains; highest levels in maize and wheat bran (JECFA 2000, EFSA 2011b)	ZEN and its metabolites interact with α- and β-estrogen receptors and endocrine disruptors (JECFA 2000, EFSA 2011b)	PMTDI 0.5 μg/kg BW/day for ZEN, recommended that the total intake of ZEN and its metabolites should not exceed the PMTDI (JECFA 2000); TDI 0.25 μg/kg BW/d for ZEN (EFSA 2011b)
Patulin (PAT)	*Byssochlamys* spp., *Penicillium* spp*.*, *Aspergillus* spp. (IARC 1986; JECFA 1996)	Many fruits, strawberries, tomatoes, olives, and cereals (IARC 1986; JECFA 1996)	Gastrointestinal ulceration; immunotoxicity and neurotoxicity in animals; genotoxic (JECFA 1996); inadequate evidence of carcinogenicity in animals, not classifiable as to its carcinogenicity to humans (IARC 1986)	PMTDI 0.4 μg/kg BW/day (JECFA 1996)
Ergot alkaloids	*Claviceps* spp., in Europe mostly *C. purpurea* (EFSA 2012, BfR 2004)	True grasses; most important on cereals (rye, wheat, triticale, barley, millet, and oats) (EFSA 2012, BfR 2004)	Interact with neurotransmitter receptors; acute toxicity: convulsive neurotroxicity, uterine hemorrhage, and abortions; chronic toxicity: vasoconstriction with ischemia and necrosis of extremities (ergotism) (EFSA 2012, BfR 2004)	Various EAs seem to have similar toxic potency; group ARfD 1 μg/kg BW/day and group TDI 0.6 μg/kg BW/day; both apply to the sum of EAs (EFSA 2012)

*PTWI* provisional tolerable weekly intake, *PMTDI* provisional maximum tolerable daily intake, *TDI* tolerable daily intake, *ARfD* acute reference dose (for 1-day exposure)

Harmful effects of mycotoxin-contaminated food can be avoided by (i) preventing contamination, (ii) removing contaminated material from the food commodity, (iii) mitigating mycotoxin content in food, and (iv) treating exposed individuals. In some commodities, only part of the harvest enters the food chain. Selection of charges with low mycotoxin levels for consumption while using the remainder for feed and energy production would reduce the exposure of consumers to mycotoxins. Unfortunately, this is only possible in a few commodities and, even there, production systems targeting food markets, feed manufacturing, and energy production are often so specialized that they cannot replace each other. The first priority therefore remains prevention of toxin accumulation directly on the field (preharvest) or thereafter (transport and storage) (Kabak et al. 2006; Choudhari and Kumari 2010). A variety of agricultural practices, e.g., growing resistant crop varieties, crop rotation, soil tillage, chemical and biological control of plant diseases, and insect control are available to minimize mycotoxin production on the field (Edwards 2004; Munkvold 2014; Mesterhazy 2014; Alberts et al. 2016). Proper harvest and storage conditions are crucial to prevent fungal growth and mycotoxin accumulation in harvested commodities (Jacobsen 2014). Unfortunately, preharvest measures do not guarantee the absence of mycotoxins in food or feed. Food processing can impact mycotoxins in raw material by (i) physical removal, (ii) chemical transformation which can result in metabolites of lower or higher toxicity, (iii) release from masked or entrapped forms which may increase bioavailability, (iv) enzymatic detoxification, and (v) adsorption to solid surfaces. Physical and chemical mechanisms reducing mycotoxin content often act together in the same food processing step. For instance, sulfur dioxide used in corn wet milling to ease the separation of germs, proteins, and starch possesses potential for chemical detoxification. Reduction of mycotoxin contamination was documented for cleaning; milling; brewing; fermentation; cooking; baking; frying; roasting; flaking; alkaline cooking; nixtamalization (soaking, cooking in an alkaline solution, and hulling of grains); and extrusion. Concentrations of some mycotoxins can be reduced substantially while others, such as DON, are relatively resistant to degradation (Milani and Maleki 2014; Karlovsky 2011). Detoxification of grain mycotoxins during food processing has recently been reviewed (Kaushik 2015). As the last resort, consumers can be prophylactically treated with binders in areas of chronically high aflatoxin exposure (Afriyie-Gyawu et al. 2008; Wang et al. 2008).

The following terms are used to describe the outcome of mitigation treatments throughout this article: *removal* of mycotoxins from raw materials and/or finished products, *transformation* (modification of the chemical structure of the molecule), *detoxification* (transformation which reduced the toxicity), and *decontamination* (removal or detoxification/inactivation). Effective decontamination should be irreversible, modified forms of mycotoxins should be affected together with parent compounds, the products should be non-toxic, and the food should retain its nutritive value and remain palatable (Milani and Maleki 2014). Processing procedures, agents, and microorganisms must be allowed for use in food (Codex Alimentarius, 2015). The interested reader is also referred to European Commission Regulation 2015/786, defining acceptability criteria for detoxification processes applied to products intended for animal feed (EC 2015). These criteria may serve as a model for the assessment of mycotoxin detoxification technologies in food processing. Compliance of a given detoxification process with those criteria will be assessed by the European Food Safety Authority (EFSA).

In this review, conventional food processing affecting mycotoxins as well as processes dedicated to decontamination are covered. Applications of the techniques to selected commodities are presented for illustration, knowledge gaps are outlined, and recommendations for prioritizating mitigation actions and further research are given.

## Physical processing methods

### Sorting

**Fig. 2:**
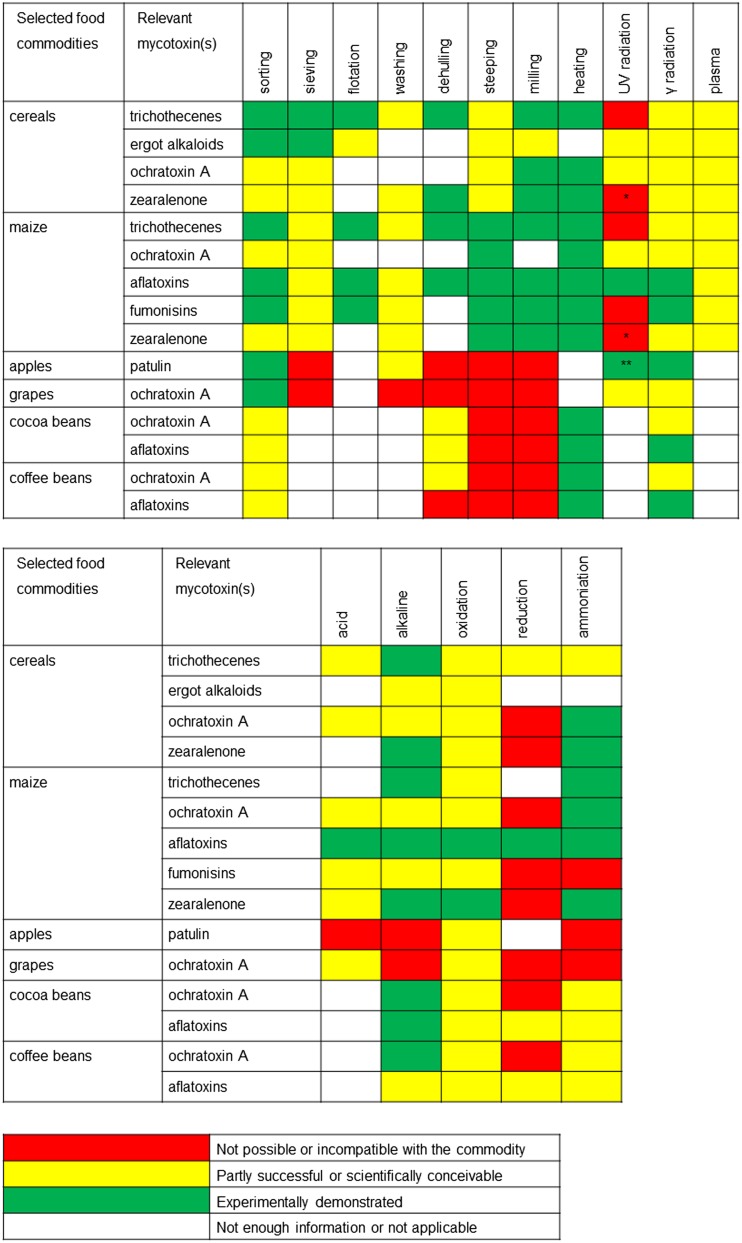
Summary of physical and chemical processes applicable to food commodities in order to mitigate targeted mycotoxins. *Conversion to a more estrogenic *cis*-form. **Experimentally demonstrated on apple juice

Unprocessed cereals in bulk trading often contain dust and admixtures. Broken and damaged kernels usually contain most of mycotoxin contamination (Johansson et al. 2006) though they constitute only 3–6 % of the bulk load (Whitaker et al. 2003). The first processing of agricultural goods after harvest often involves sorting, washing, or milling (Grenier et al. 2014). Figure 2 summarizes the use of these techniques. Sorting machines based on particle weight and size are in use since the end of the nineteenth century (Mayer 1898). Originally, grains were sorted in bulk using centrifugation force and flotation in air flow. In the 1960s, optical sorting was established. The operation principle is to direct streams of grains along an array of optical sensors. When a grain differing in color is detected, the detector triggers a magnetic valve and a jet of pressurized air removes the kernel from the stream (Fraenkel 1962). This principle is still used today. Contemporary grain sorters have a throughput of dozens of tons grain per hour.

Aflatoxin contamination is usually heterogeneous so that separating damaged kernels can effectively reduce contamination (Kabak et al. 2006). Grain sorting using UV light illumination for aflatoxin reduction is common. The observed bright greenish-yellow fluorescence (BGYF) does not originate from aflatoxins but from a kojic acid derivative following reaction with endogenous peroxidase. In dried commodities, peroxidase is inactivated and the BGYF method does not work. The quick and easy “black light test” may therefore result in both false positive and false negative findings (Bothast and Hesseltine 1975). Although the test is not as reliable as originally hoped (Doster and Michailides 1998), it is widely used, e.g., by Turkish companies exporting dry figs and nuts to the EU. As an audition by the Food and Veterinary Office of the EU confirmed, the efficiency of sorting is regularly verified by laboratory analysis (EC 2013).

Distribution of ergot alkaloids (EAs) is even more heterogeneous than aflatoxins because intermediate contamination does not exist at a single-kernel level. Sclerotia loaded with EAs are efficiently removed from rye by opto-electronic sorting (Young et al. 1983; Miedaner and Geiger 2015).

Because infection with *Fusarium verticillioides* often does not cause symptoms (Munkvold and Desjardins 1988) and correlation between fumonisin content and symptoms is weak (Afolabi et al. 2007), grain sorting might not reduce fumonisin content efficiently though successful attempts have been reported (Pearson et al. 2004). Mycotoxins accumulating without visible symptoms pose a limit to optical sorting as a mycotoxin mitigation strategy. This may explain why no reduction of aflatoxin content by sorting was found in a recent study (Mutiga et al. 2014).

### Sieving cleaning

Removing kernels with extensive mold growth, broken kernels, and fine materials such as dirt and debris can be achieved by sieve cleaning, which significantly lowers total mycotoxin contamination. Removal of EAs from wheat grains by sieving has been used as a plant quarantine treatment (Muthaiyan 2009). After sieving off corn screenings, it was determined that intact kernels contained about 10 times less fumonisins than broken corn kernels or smaller parts (Murphy et al. 1993). Particles passing through a 3-mm sieve usually constitute 5–20 % of a sample by mass, but contain 26–69 % of fumonisins (Sydenham et al. 1994). Removing broken kernels and smaller parts from maize reduced DON and ZEN contamination by around 70–80 %; however, up to 69 % of the total maize was rejected as well (Trenholm et al. 1991). Losses of barley or wheat were lower with 34 and 55 % for comparable mycotoxin reduction.

### Flotation and density segregation

The different physical properties of mold-damaged kernels compared to non-damaged kernels can be exploited to separate them by density segregation or by fractionation on gravity tables. The damage to the kernels is caused by different fungi, rendering this techniques sensitive to the overall fungal contamination, rather than to specific toxins. Ergot containing EAs can be efficiently separated from rye grains by flotation in NaCl solution (Plante and Sutherland 1944). Removal of corn buoyant in water reduced aflatoxin levels by 60 %, at a mass loss of 22 % (Huff 1980). Using a 30 % sucrose solution, 87 % aflatoxin reduction was achieved by the removal of 50 % of the material (Huff 1980). Flotation on a saturated sodium chloride solution removed not only 3 % of the kernels but also 74 % of the total aflatoxin content in maize (Huff and Hagler 1985). Likewise, the removal of kernels floating on both water and 30 % sucrose reduced more than 53 % DON in maize and above 68 % in wheat. The same procedure basically removed all ZEN in the tested samples (Huff and Hagler 1985). In a later study, fumonisin reduction of 86 % was achieved by removing maize kernels buoyant in saturated brine, with about 20 % material loss (Shetty and Bhat 1999).

### Washing

Water-soluble mycotoxins can be partly washed from the surface of grains. ZEN is barely water-soluble, but well soluble in alkaline solutions. Therefore, sodium carbonate solutions are often used as an alternative to improve the effectiveness of washing steps. Washing barley and corn three times in distilled water reduced the DON content by 65–69 %, while ZEN concentrations were reduced by 2–61 %. Using 1 mol/l sodium carbonate solution for the first wash step reduced DON by 72–75 % and ZEN by 80–87 % (Trenholm et al. 1992). In a similar study, the concentration of both toxins was reduced by 44 % in corn by a single rinsing step with water. Additional soaking of the material in a 0.1 mol/l aqueous sodium carbonate solution further reduced DON and ZEN concentrations by 35 % (Rotter et al. 1995). As the soaking step took a full day, this technique should already be regarded as a chemical processing step (see next chapter). Washing and buoyancy techniques both suffer from the shortcoming that the grain must be dried after treatment before it can be stored. In order to ensure an efficient washing of contaminated commodities with water or water-based solutions, parameters such as partition coefficient or solubility should be considered and are given in Table 2.

**Table 2:** Hydrophobicity (log P) and solubility in water (in mg/l) of selected mycotoxins. Given data were gathered from databases using predictive tools

Name	predicted log P*	Predicted solubility in water (mg/l)*	Entry number in the toxin and toxin target database (T3DB)
Ochratoxin A	3.66	25.6	http://www.t3db.ca/toxins/T3D3605
Zearalenone	3.04	117	http://www.t3db.ca/toxins/T3D3665
Ergotamine	2.95	223	http://www.t3db.ca/toxins/T3D2460
T-2 toxin	1.95	347	http://www.t3db.ca/toxins/T3D3664
HT-2 toxin	1.32	1120	http://www.t3db.ca/toxins/T3D3673
Aflatoxin B_1_	1.73	233	http://www.t3db.ca/toxins/T3D3598
Aflatoxin B_2_	1.63	392	http://www.t3db.ca/toxins/T3D3669
Aflatoxin G_1_	1.81	424	http://www.t3db.ca/toxins/T3D3670
Aflatoxin M_1_	1.21	994	http://www.t3db.ca/toxins/T3D3666
Citrinin	1.23	1160	http://www.t3db.ca/toxins/T3D3597
Patulin	−0.27	163,000	http://www.t3db.ca/toxins/T3D3661
Deoxynivalenol	−0.76	36,000	http://www.t3db.ca/toxins/T3D3668
Nivalenol	−0.79	64,600	http://www.t3db.ca/toxins/T3D3674
Fumonisin B_1_	−0.81	> 20,000**	http://www.t3db.ca/toxins/T3D3603
Fumonisin B_2_	−0.28	> 20,000**	http://www.t3db.ca/toxins/T3D3697

*Data from ALOGPS 2.0 (http://www.t3db.ca/toxins)

**Experimental data from the US National Toxicology Program (NTP 2000)

### Dehulling

The outer layers of grain are removed by dehulling techniques, which are formerly composed of an indispensable processing step prior to grinding. Limitation of fungal colonization and mycotoxin accumulation to surface layers of the kernel are prerequisites for the success of dehulling in mycotoxin content reduction (Vučković et al. 2013). This condition is fulfilled for aflatoxins in maize, dehulling of maize can therefore remove up to 93 % of aflatoxins (Siwela et al. 2005). During the preparation of muthokoi, a traditional dehulled maize dish in Kenya, aflatoxin content was reduced by 46.6 % (Mutungi et al. 2008), leading to significantly lower dietary exposure due to maize meal and muthokoi consumption as compared to the consumption of entire kernels (Kilonzo et al. 2014).

### Steeping

This is the first step in wet milling of maize and involves soaking maize for 36–50 h at 50 °C in water containing 0.1 to 0.2 % SO_2_ to facilitate germ separation and breaking down of protein matrix. Adding SO_2_ also promotes lactic acid production which can be regarded as a chemical treatment from this review’s perspective. Half of the aflatoxin content of corn was found in the steep liquor (Aly 2002). Fumonisins, which are highly polar, migrate from kernels into steeping water (Canela et al. 1996)_._ Pujol et al. (1999) reported that steeping corn kernels in 0.2 % solution of SO_2_ at 60 °C for 6 h was effective in reducing FB1. OTA was distributed equally between solubles and corn grits (Wood 1982). Steeping sorghum grains in 0.2 % NaOH reduced the concentration of aflatoxins, fumonisins, ZEN, and DON under detectable levels (Lefyedi and Taylor 2006).

### Milling

After milling small-grain cereals, high mycotoxin levels are found in bran, such as DON, while finished flour is contaminated to a much lower degree (Cheli et al. 2013; Tibola et al. 2015). Spatial distribution of DON, ZEN, and their masked forms in wheat milling fractions was studied by Schwake-Anduschus et al. (2015). ZEN was concentrated in fiber-rich parts of grains DON contaminated all fractions equally, showing that the efficiency of milling as a mycotoxin mitigation strategy is limited to commodity/mycotoxin pairs in which mycotoxins are enriched in fractions that can be removed from processing.

Wet milling of maize results in germ (further processed into germ oil), starch, and gluten. Different mycotoxins accumulate in different fractions except for the starch fraction in which all mycotoxins are reduced below a level of concern. 40–50 % of aflatoxins moved from corn into steep water in wet milling, 28–38 % remained in the fiber fraction, 11–17 % in the gluten fraction, 6–11 % in the germ, and only 1 % in starch (Yahl et al. 1971; Bennett and Anderson 1978). Fumonisins are partly dissolved in steep water. At very high contamination levels, significant amounts of fumonisins remained in gluten and fiber. Germ fractions are less affected and starch is virtually free of fumonisins (Bennett et al. 1996). Two thirds of T-2 toxin were removed by steep and process water during wet milling of maize, starch contained 4 % of the toxin and the remainder was evenly distributed between germ, gluten, and fiber (Collins and Rosen 1981). OTA present in steeped corn (see earlier) went into process water solubles and corn grits in almost equal amounts while only 4 % were transferred to the germ (Wood 1982).

Dry milling of maize grain leads to concentration of mycotoxins in germ and bran fractions (Bullerman and Bianchini 2007). Aflatoxins are concentrated in germ fraction to a higher degree than fumonisins (Pietri et al. 2009). Increased concentrations of 16 *Fusarium* spp. mycotoxins in bran and germ as compared to whole grain were reported for dry-milled maize by Schollenberger et al. (2008). As expected, apolar ZEN was mainly found in germ and bran fractions after dry milling (Bennett et al. 1976). Milling results in redistribution of the ergot sclerotia with EAs among milling fractions (EFSA 2012).

### Heat treatment

The time/temperature combination undoubtedly remains one of the most important interventions by which industrial processing can affect the mycotoxin content in a finished food product. Most mycotoxins are chemically and thermally stable though. While conventional food preparation with temperatures up to 100 °C have little effect on most mycotoxins, higher temperatures used in frying, roasting, toasting, and extrusion might reduce mycotoxin contamination.

Aflatoxins can be reduced by extrusion by 50–80 %, depending on grain moisture and temperature (Bullerman and Bianchini 2007). Alkaline treatment (see next chapter) can increase the efficacy of this process. Similar results were achieved for peanut meal, when extrusion alone reduced aflatoxins by 23–66 % and up to 87 % in the presence of ammonium hydroxide (Cheftel 1989). Roasting can reduce the levels of aflatoxins by 50–70 % in peanuts and pecans and by 40–80 % in maize (Conway et al. 1978). Pure aflatoxin B_1_ (AFB_1_) was destroyed by temperatures above 160 °C; soybean matrix accelerated the process (Raters and Matissek 2008). Roasting can reduce the content of OTA in coffee beans by up to 97 %, depending on the temperature and particle size (Oliveira et al. 2013). Degradation of OTA in wheat by heating (Boudra et al. 1995) and extrusion (Scudamore et al. 2004) was less efficient. Thermal degradation products of OTA are 14-(R)-ochratoxin A, 14-decarboxy-ochratoxin A and ochratoxin alpha amide, all of which have reduced toxicity (Cramer et al. 2008; Bittner et al. 2015). Cazzaniga et al. (2001) reported drastic reduction of the level of DON under all investigated conditions but other labs found moderate effects depending on the conditions (Wu et al. 2011) or no reduction of DON and nivalenol (NIV) (Scudamore et al. 2008). Extrusion cooking of maize grits contaminated with ZEN reduced the toxin content by 65–83 % (Ryu et al. 1999). Extrusion or roasting was also effective in reducing fumonisins in maize grits by 34–95 % (Bullerman and Bianchini 2007). Increased temperature, decreased screw speed, and glucose addition resulted in higher reduction rates during extrusion. Thermal treatment always involves transformation reactions. Fumonisin in corn extruded with glucose (Bullerman and Bianchini 2007) yielded *N*-(1-deoxy-d-fructos-1-yl)-fumonisin B_1_, a compound less toxic than fumonisin B_1_ to rats (Hahn et al. 2015). Citrinin (CIT) could also be efficiently degraded by heating (Trivede et al. 1992). EAs are partly degraded and epimerized during bread baking; the ratio between epimers shifts towards the -inine forms (EFSA 2012; Merkel et al. 2012).

### Irradiation

Irradiation may be an approach for removing mycotoxins on an industrial scale, providing in fact energy to both food constituents and contaminants: reactions occur and change the molecular structure of food constituents. Non-ionizing (solar, UV, microwave) and ionizing (gamma) radiations can reduce or eliminate pathogenic microorganisms, but partly also mycotoxins in food.

Photodegradation of aflatoxins in cereals has been found to decrease toxin levels by about 40 % after 3 h and up to 75 % after 30 h of direct sunlight (Herzallah et al. 2008). The same authors found sunlight to be more effective than 10 min of microwave heating (32 % reduction) or gamma-irradiation with 25 kGy (43 % reduction). In another study, peanuts, pistachios, rice, and corn were irradiated with gamma radiation (Ghanem et al. 2008). At higher doses, the aflatoxin reduction was pronounced, reaching 59–88 % at 10 kGy. However, the most recent study showed aflatoxin reductions of only 11–21 % at 15 kGy (di Stefano et al. 2014). The same study reported the reduction of OTA in almonds to 24 % by gamma radiation at 15 kGy. This number is in good agreement with fumonisin reduction in maize, which was found to be about 20 % after a dose of 15 kGy (Visconti et al. 1996). Microwave treatment (and convection heat) was partially successful in lowering DON levels in naturally contaminated maize, with greatest effect occurring at the highest temperatures. With final temperatures of 150–175 °C, a 40 % reduction was achieved (Young 1986). This reduction can be explained by the formation of several nor-DONs, which are far less cytotoxic than DON (Bretz et al. 2006). Exposure of vegetable oil contaminated with ZEN to sunlight through common glass bottles caused isomerization of natural *trans*-ZEN into *cis*-ZEN with a conversion of up to 90 % (Köppen et al. 2012). UV light is very effective in removal of PAT in apple juice and cider. Evaluation of the reduction of PAT in apple juice at different wavelengths in the UVC range showed that 222 nm was most suitable (Zhu et al. 2014). At an initial level of about 1000 μg/l, the UV exposure was successful in reducing PAT levels by 5 to 73 %, depending on the number of passes (Assatarakul et al. 2012). UV exposure, however, affected the taste of apple juice and cider.

### Cold plasma

Cold plasma has strong antimicrobial effects and can be used to sterilize fragile or temperature-sensitive surfaces, such as food. A recent review on the use of plasma for food processing (Schlüter et al. 2013) highlighted the potential of this new technique that at the same time demands cautious use. No investigation on potential formation of toxic compounds by plasma treatment has been conducted yet. The authors concluded that plasma-treated products have to be assessed on a case by case basis for the time being.

Low-pressure cold plasma destroyed up to 50 % of alfatoxins on nut surfaces (Basaran et al. 2008). The effect of atmospheric pressure argon cold plasma on spores and mycotoxin production of *Aspergillus niger* contaminating date palm fruits was recently evaluated (Ouf et al. 2015). After treatment for 9 min, all fungal spores were killed, OTA and fumonisin B_2_ contents dropped from 25 and 6 μg/100 mm^2^, respectively, below the limits of detection. Cold plasma generated by atmospheric dielectric barrier discharge in a direct and remote mode with synthetic air as working gas reduced the concentration of DON and ZEN in thin layers from 100 μg/ml to a few micrograms per milliliter (ten Bosch et al. 2014).

### Mycotoxin binders

Mycotoxin binders are a physical technique used for feed decontamination (Jans et al. 2014) that principally can also be used in human intervention. Activated charcoal was used to remove patulin from naturally contaminated cider and bentonite removed AFM_1_ from naturally contaminated milk (Doyle et al. 1982). De Nijs et al. (2012) discussed the efficiency of mycotoxin mitigation and food safety aspects of such techniques. The efficiency of binders in mitigating adverse effects of aflatoxins in food was demonstrated in a randomized and double-blinded clinical trial (Wang et al. 2005; Afriyie-Gyawu et al. 2008; Wang et al. 2008). These are the only reports on the use of such techniques in food so far.

## Chemical processing methods

Many studies investigated chemical food processing methods for their suitability to destroy or inactivate mycotoxins. It should be noted that chemical treatment for the purpose of detoxification or decontamination is not authorized within the EU for commodities destined for human food; dedicated mitigation treatments would therefore require regulatory approval. Chemicals transform mycotoxins into other compounds, the toxicity of which must be assessed. It is furthermore crucial that the treatment does not impair nutritional quality, texture, or flavor of food. Criteria recently defined for the approval of detoxification techniques for feeds (EC 2015) may serve as a model for the development of corresponding regulations for food.

Common food processing technologies may reduce mycotoxin content as a side effect of accompanying chemical processes. Codex Alimentarius provides a list of such chemicals (Codex Alimentarius General Standard for Food Additives) and national legislations regulate their use. In this section, the effect of chemical treatments on mycotoxins in food will be reviewed regardless of whether the treatments are part of standard food processing or they have been studied as dedicated detoxification methods. The use of chemicals in combination with physical treatments, described in the previous section, may increases the efficacy of mycotoxin degradation. Chemical agents for mycotoxin detoxification can be applied by mixing, packing, fumigation or immersion.

### Acid treatment

Most of the known mycotoxins are resistant against weak acids, as reviewed by Müller (1983). However, treatment of aflatoxins with strong acids destroyed the biological activity of AFB_1_ and AFG_1_ by converting them to hemiacetal forms AFB_2a_ and AFG_2a_, respectively (Ciegler and Peterson 1968; Dutton and Heathcote 1968). Treatment with HCl (pH 2) reduced AFB_1_ levels by 19 % within 24 h (Doyle et al. 1982). In the presence of acetic anhydride and hydrochloric acid, the reaction proceeds further to give the acetoxy derivative. Similar adducts of AFB_1_ and AFG_1_ are formed with formic acid-thionyl chloride, acetic acid-thionyl chloride, and trifluoroacetic acid. Aiko et al. (2016) treated aflatoxins with diluted acetic acid, citric acid, and lactic acid under conditions simulating cooking. Lactic acid was most efficient, converting AFB_1_ into AFB_2_ (traces) and AFB_2a_ (major product). Apart from detoxifying aflatoxins, small carboxylic acids inhibit mold growth, and are therefore used as preservatives.

### Treatment with bases

Aflatoxins are unstable under alkaline conditions (Kiermeier and Ruffer 1974; Itoh et al. 1980), the first step of degradation being the opening of the lactone ring. Because this step is reversible, it is important to allow the reaction to proceed to completion. The degradation of aflatoxins in groundnut and cottonseed meal as well as in corn by sodium hydroxide and other alkaline reagents (Ca(OH)_2_, Na_2_CO_3_, Na_3_PO_4_, methylamine, ethylene-diamine, ethanolamine) has been reviewed by Müller (1983). In most cases, a partial detoxification was achieved. Degradation of aflatoxins using ammonia has been extensively studied and proved effective in laboratory experiments as well as in field trials effective (Müller 1983; Park et al. 1988). Ammoniation of AFB_1_ resulted in two major breakdown products which retained the difuran moiety but lost the lactone ring: aflatoxin D_1_ and D_2_ [4-hydroxy-6-methoxy-3a,8a-dihydrofuro[2,3]benzofuran] (Cucullu et al. 1976). Ammoniation decreased aflatoxin levels in maize by more than 75 % (Park et al. 1988) and completely decomposed OTA in maize, wheat, and barley (Chełkowski et al. 1981). Ammoniation can reduce aflatoxin concentration by more than 99 % (Chełkowski et al. 1981; Masri et al. 1969; Brekke et al. 1977). The efficacy of the process depends on the temperature, pressure, moisture, duration, and the substrate (Weng et al. 1994). Park et al. (1988) reviewed the decontamination of aflatoxins by ammoniation and concluded that the results demonstrate overwhelming support for the efficacy and safety of ammoniation as a practical solution to aflatoxin detoxification in oilseed meals intended for animal feeding. In spite of three decades of encouraging research, ammoniation of food commodities has, to our knowledge, not been approved in any country so far.

The instability of rubratoxin and cyclochlorotin under alkaline conditions was shown by Moss (1971) and Ishikawa et al. (1970), respectively. Chełkowski et al. (1981) demonstrated reduction of the content of penicillic acid, CIT, OTA, ZEN, and AFB_1_ after treatment with 2 % aqueous NH_3_. PAT was unstable already at pH 7, while at pH 8, it was degraded completely after 190 h (Brackett and Marth 1979). Bennett et al. (1980) described 80 % reduction of ZEN in spiked corn grits and 64 % reduction in naturally contaminated corn after treatment with 3 % NH_3_ at 50 °C for 16 h.

Treatment of FB_1_-contaminated corn with Ca(OH)_2_, simulating nixtamalization (soaking/cooking in an alkaline solution), completely hydrolyzed FB_1_ but the toxicity of the products in brine shrimp assay was only partly reduced (Park et al. 1996). Ammoniation reduces the concentration of FB_1_ in wheat by 79 % (Park et al. 1992) but was inefficient in corn according to Norred et al. (1991). Toxicity of the products of alkaline hydrolysis of fumonisins varies considerably among species (see enzymatic hydrolysis of fumonisins in the following section for details). Until these effects are fully understood, alkaline treatment of fumonisin-containing food ingredients should be avoided.

### Use of oxidizing agents

The oxidation of aflatoxin appears to be primarily an addition directed towards the double bond of the terminal furan ring, followed by subsequent reactions involving the phenol formed on opening the lactone ring. It was well-known that aflatoxins such as AFB_1_, AFG_1_, and AFM_1_, which had a terminal double bond in the dihydrofuran ring were more susceptible to attack by ozone (O_3_) and other oxidizing agents than AFB_2_, AFG_2_, and AFM_2_, which lack this double bond (McKenzie et al. 1997). It is postulated that ozone reacts with the C8–C9 double bond of the furan ring of aflatoxin through electrophilic attack, causing the formation of primary ozonides followed by rearrangement into derivatives such as aldehydes, ketones, and organic acids (Proctor et al. 2004). Studies have demonstrated that ozone is able to degrade aflatoxins in different commodities (Dwarakanath et al. 1968) and in aqueous solutions (Maeba et al. 1988). AFB_1_ and AFG_1_ were sensitive to ozone and easily degraded with 1.1 mg/l of ozone within 5 min at room temperature. On the other hand, aflatoxins B_2_ (AFB_2_) and G_2_ (AFG_2_) were rather resistant to ozone, requiring 50–60 min to degrade them completely with 34.3 mg/l of ozone. These forms require longer exposure for detoxification; a possible mechanism is opening of the lactone ring (Samarajeewa et al. 1990).

Ozone was reported to reduce AFB_1_ and AFG_1_ levels by 77 and 80 %, respectively, in peanuts after treatment at 75 °C for 10 min, while the maximum degradation of 51 % was achieved for AFB_2_ and AFG_2_ regardless of the exposure time (Proctor et al. 2004). In another study, the reductions of AFB_1_ in paprika were 80 and 93 % after exposures to 33 and 66 mg/l O_3_ for 60 min, respectively (Inan et al. 2007). Ozone degradation has been shown to be effective also against other mycotoxins such as DON (Young 1986, Young et al. 1986) and moniliformin (Zhang and Li 1994). A highly concentrated ozone produced by an electrochemical method (Rogers et al. 1992) was able to degrade and detoxify several mycotoxins in vitro, including aflatoxins, cyclopiazonic acid, OTA, PAT, secalonic acid D, and ZEN after treatment with O_3_ at 10 % for 15 s (McKenzie et al. 1997).

Hydrogen peroxide, H_2_O_2_, was used on a commercial scale to detoxify aflatoxins. Treatment of figs with H_2_O_2_ at 0.2 % caused a 66 % reduction in AFB_1_ levels following 72-h storage (Altug et al. 1990). Hydrogen peroxide reduced aflatoxin concentrations in corn (Chakrabarti 1981), peanut meal (Sreenivasamurthy et al. 1967), and milk (Applebaum and Marth 1982). The concentration and the toxicity of ZEN decreased after treatment with aqueous solution of H_2_O_2_ (Lasztity et al. 1977). The efficiency of H_2_O_2_ for destruction of ZEN in contaminated corn was found to dependent upon the concentration of H_2_O_2_, temperature, and period of exposure (Abd Alla 1997). CIT was completely detoxified by 0.05 % H_2_O_2_ after 30 min whereas OTA was resistant to this treatment (Fouler et al. 1994).

Matsuura et al. (1979) reported that ZEN is destroyed by oxidation with ammonium persulfate. Natarajan et al. (1974) showed that sodium hypochlorite concentration and pH, but not temperature and time, affected the destruction of aflatoxins in peanut protein. Aflatoxin degradation by sodium hypochlorite was compared with the effect of sodium hydroxide and ammonium hydroxide (Draughon and Childs 1982). All three treatments significantly reduced fluorescence but the survival of brine shrimp has not always increased. The mutagenic and cancerogenic aflatoxin B_1_-2,3-dichloride may be formed using sodium hypochlorite, although this can be avoided by adding acetone (Castegnaro et al. 1981). Aflatoxins were removed completely from rice meal treated with 16.5 % NaCl and 1 % NaOCl for 24 h (Okonko and Nwokolo 1978).

### Treatment with reducing agents

Sodium bisulfite (NaHSO_3_) was shown to destroy mycotoxins, primarily AFB_1_ in maize (Doyle et al. 1982) and dried figs (Altug et al. 1990). Dried fig fruits were spiked to contain 250 μg/kg AFB_1_ and treated with sodium bisulfite (1 % in the aqueous phase). This treatment caused 28 % of added toxin to degrade within 72 h at 25 °C. When 0.2 % H_2_O_2_ were added 10 min before the bisulfite treatment, 65 % of AFB_1_ were degraded in 72 h. Heating bisulfite-treated samples at 45 to 65 °C for 1 h caused up to 68 % of added AFB_1_ to be degraded. Promising results have been achieved in AFB_1_, AFG_1_, and AFM_1_ detoxification using sodium bisulfite (Doyle and Marth 1978a, b; Moerck et al. 1980; Hagler et al. 1982; Yagen et al. 1989). Moerck et al. (1980) and Hagler et al. (1982) demonstrated efficient destruction of low and high levels of aflatoxin in corn. The decontaminated corn had improved color, better palatability, better handling properties, improved economics, and the residual bisulfite was a permitted food additive. Yagen et al. (1989) established the structure of aflatoxin B_1_S as 15α-sodium sulfonate of AFB_1_. The formation of AFB_1_ products substituted at the 15th position only is unprecedented and implies an unusual mechanism. The completeness of the reaction and the water solubility of aflatoxin B_1_S supports the use of bisulfite as a promising method to mitigate AFB_1_ and AFG_1_.

NaHSO_3_ solutions reduced DON in contaminated maize (4.4 mg/kg) by 85 % after 18 h at 80 °C (Young et al. 1987). Sodium metabisulfite at 10 g/kg was reported to overcome the depressing effect of DON on feed intake in piglets (Dänicke et al. 2005). Reduction of DON in animal feed by treatment with sodium bisulfite and sodium metabisulfite has been demonstrated in several studies. The treatment leads to the formation of less toxic DON sulfonate; a review is available (Dänicke et al. 2012). Later, it was discovered that the use of different sulfur reagents for reduction of DON yielded three different DON sulfonates with the same mass and molecular formula (Schwartz et al. 2013). DON sulfonate 1 is characterized by loss of the epoxide group, and DON sulfonate 2 by formation of a hemiketal. DON sulfonate 3 is an equilibrating mixture of two isomers, a ketone and a hemiketal. Rapid formation of DON sulfonates 1 and 2 occurs at alkaline pH, slow formation of DON sulfonate 3 takes place at acidic pH, irrespective of the sulfur reagent used. Whereas DON sulfonates 1 and 2 are stable across a broad pH range, DON sulfonate 3 decomposes to DON and DON sulfonates 1 and 2 at alkaline pH (Schwartz et al. 2013).

### Treatment with food ingredients and medical plants

Certain spices, herbs, and other ingredients used in food production and home cooking were shown to detoxify mycotoxins. Incubation with extract of ajwan (carom), used as a spice in Asian cooking, destroyed aflatoxins (Velazhahan et al. 2010). Extracts of medicinal plants *Ocimum tenuiflorum* detoxified aflatoxins even at room temperature (Panda and Mehta 2013) and aqueous extracts of vasaka leaves (*Adhatoda vasica*) degraded AFB_1_ completely after 24 h at 37 °C (Vijayanandraj et al. 2014). Detoxification of aflatoxins by Indian spices and herbs was recently reviewed (Aiko and Mehta 2015). Reducing sugars such as D-glucose and D-fructose blocked the primary amino group of FB_1_, after incubation at 65 °C for 48 h, preventing FB_1_-induced toxicity on cell tissue cultures on rats and swine (Fernandez-Surumay et al. 2005).

## Enzymatic detoxification

### Distinguishing features of enzymatic detoxification

Enzymatic catalysis takes a unique position among activities potentially suitable to detoxify mycotoxins. A distinguishing feature of enzymatic detoxification is its specificity. Notable exceptions are laccases and peroxidases, which have been tested for degradation of mycotoxins (Alberts et al. 2009; Wang et al. 2011) though they modify a wide range of substrates and may thus destroy valuable food components. The potential of enzymatic activities for the detoxification of mycotoxins in general was recently reviewed (Vanhoutte et al. 2016) but only a single short review is available on the potentical of enzymatic detoxification of mycotoxins in food production (Karlovsky 2014).

Enzymes are proteins and, when used in food processing, may cause allergy. However investigation of the allergenic potential is part of the documentation required for the approval of enzymes as additives or processing aids (EFSA 2009; JECFA 2001b). No allergic reaction to current food enzymes has been reported so far, indicating that enzymes are of limited concern regarding food allergies (Bindslev-Jensen et al. 2006). Because of their specificity and favorable toxicological profile, enzymes possess a yet unexplored potential to detoxify organic contaminants in food. A recent application pointing the way is the use of recombinant asparaginase to prevent formation of acrylamide in bread (Hong et al. 2014). No enzyme has so far been authorized in the EU for the reduction of mycotoxin contamination in food.

### Intentional use of enzymes to detoxify mycotoxins in food production

Enzymes are used extensively as processing aids. For instance, recombinant aspartic protease chymosin is an alternative to rennet in the manufacturing of cheese (Teuber 1993) and industrial enzymes of five classes are used in bread making (Whitehurst and van Oort 2010). Malting and brewing are further examples of processes that would benefit from the use of enzymes detoxifying mycotoxins. Beer is commonly contaminated with DON (see section “Ethanol and beer”). Adding enzymes detoxifying DON to amylases, glucanases, proteases, and other enzymes used in beer production (Whitehurst and van Oort 2010) is compatible with brewing technologies. Unfortunately, enzymes suitable for irreversible detoxification of DON are not available yet. Promising new detoxification activities have been identified (Ito et al. 2013; He et al. 2015); it remains to be seen whether enzymes responsible for these activities are suitable for industrial production.

DON is a major mycotoxin contaminating wheat. Because the same mycotoxin occurs in beer and wheat flour, enzymes envisaged for the detoxification of DON in beer might be suitable for bakery products, too. Such an enzyme could be added to wheat flour together with recombinant xylanases, proteases, amylases, and other enzymes commonly used in the process.

Another example of the potential of industrial enzymes to reduce exposure to mycotoxins is the detoxification of PAT. Fruit juices and particularly purees may contain PAT. Production of juices involves treatment with pectinases/arabanases, glucoamylases, and other enzymes. Enzymatic activities degrading PAT have been found in many species of bacteria and yeast (for instance, Zhu et al. 2015b). Degradation products are less toxic (Castoria et al. 2011; Zhu et al. 2015a), indicating that mitigation of PAT by enzymes is feasible. At least one of the enzymes detoxifying PAT does not require diffusible cofactors and is active in semi-purified form (Zhu et al. 2015a). Degradation of PAT can likely be combined with current enzymatic treatments used in the production of fruit juices and purees.

Widespread use of enzymes in food processing suggests that detoxification of mycotoxins by enzymatic treatment is compatible with current food technologies.

### Examples of enzymatic activities suitable for the detoxification of mycotoxins in food processing

Because fumonisins cause severe, species-specific diseases in farm animals (Voss et al. 2007) and are presumably also in humans (Isaacson 2005), extensive research on fumonisin detoxification has been carried out (Alberts et al. 2016). Enzymes detoxifying fumonisins were found in black yeast *Exophiala spinifera* (Blackwell et al. 1999) and genetically engineered maize varieties detoxifying fumonisins by enzymes of the yeast were developed (Duvick 2001). Fumonisin-detoxifying bacterium *Sphingomonas* spp*.* was characterized (Heinl et al. 2010) to provide enzymes for the decontamination in animal feeds; applications of these enzymes in food production are explicitly considered in the pertaining patent. Fumonisins are polyketides possessing an amino group and esterified with two carballylic acid residues. Microbial degradation of fumonisins is initiated by the hydrolysis of the ester bonds, which reduced the toxicity of fumonisins in pigs (Grenier et al. 2012) but not in rats (Seiferlein et al. 2007). Biochemical studies showed that hydrolyzed fumonisins might be transformed to highly toxic derivatives in vivo (Humpf et al. 1998), which might account for species differences in the toxicity of hydrolyzed fumonisins.

It was shown already in 1988 that the fungus *Clonostachys rosea (*syn. *Gliocladium roseum*) is capable to metabolize ZEN in high yield (el-Sharkawy and Abul-Hajj 1988) to a less estrogenic product. Two groups identified the *C. rosea* gene encoding a ZEN**-**specific hydrolase (Takahashi-Ando et al. 2002; Karlovsky et al. 2003). The enzyme does not require cofactors and appears suitable for food processing. Further activities detoxifying ZEN have been found in yeasts, bacteria, and fungi (Vekiru et al. 2010; Tan et al. 2014; Popiel et al. 2014).

Numerous enzymes degrading OTA have been described (Abrunhosa et al. 2010). Because OTA is an amide, many peptidases are able to hydrolyze this mycotoxin, including carboxypeptidase and chymotrypsin (Pitout 1969). Numerous lipases were shown to hydrolyze OTA, too (Stander et al. 2000). An enzyme from *A. niger* cleaves OTA into less toxic OTα and phenylalanine (Dobritzsch et al. 2014).

Enzymatic detoxification appears conceivable for any mycotoxin but a proof of concept for food processing is yet to be provided.

## Microbial decontamination

### Detoxification of mycotoxins as a side effect of fermentation

Fermentation is food processing with the help of microorganisms. Activities of bacteria and fungi used in fermentations are responsible for desired transformations of food components but hundreds of additional enzymatic activities are present in their cells, actively secreted into food matrix or released from disintegrated cells after autolysis. Some of these activities may transform mycotoxins into non-toxic products (Wolf-Hall and Schwarz 2002) but no microbial strain has been authorized so far as a processing aid targeting mycotoxins. Malting and brewing are prominent examples of technologies that may benefit from such fermentation aids. Considering how much beer per capita is consumed in industrial countries, beer contributes significantly to exposure of consumers to DON and ZEN (see section “Ethanol and beer”). Detoxification of several mycotoxins during malting has been documented, including complete loss of OTA and CIT (see section “Ethanol and beer”) and loss of most of EAs during malting and brewing (Schwarz et al. 2007) but DON survives the process (Scott 1996).

Manufacturing of many dairy products involves fermentation with lactic acid bacteria. The major mycotoxin of concern in milk is aflatoxin M_1_ (see section “Milk and other dairy products”). Detoxification of aflatoxins by lactic acid bacteria has been studied for three decades (Megalla and Mohran 1984). Unfortunately, we still do not know whether the loss of aflatoxins observed after incubation with lactic bacteria is caused by adsorption, as shown for some lactobacteria (Pierides et al. 2000; Haskard et al. 2001), or whether irreversible enzymatic transformations occurred. This question has not been rigorously addressed so far in spite of its eminent relevance for food safety.

Cultures of yeast *Saccharomyces cerevisiae* are used in wine making, brewery, and sour dough production. *S. cerevisiae* was shown to detoxify the mycotoxins PAT (Moss and Long 2002) and OTA (Petruzzi et al. 2014). Alcoholic fermentation of fruit juices destroys PAT, therefore fermented products such as cider and perry will not contain PAT (FAO/WHO 2003). Products of the transformation of ZEN by *S. cerevisiae* retain their estrogenicity (Matsuura and Yoshizawa 1985; Böswald et al. 1995). Other mycotoxins such as fumonisins and some trichothecenes are not affected by fermentation (Bothast et al. 1992; Schwartz et al. 1995).

An example of technology with potential to reduce mycotoxin content by fermentation is the production of tempeh in Indonesia. Tempeh is traditionally made of soybean fermented with *Rhizopus oligosporus*. Some current forms of tempeh contain maize and groundnuts, which are prone to contamination with aflatoxins. Interestingly, *Rhizopus* spp. strains were reported to detoxify aflatoxins (Nakazato et al. 1990). The safety of tempeh can possibly be improved by selecting starter cultures that detoxify aflatoxins without any modification of the technology.

### Detoxification of mycotoxins by pure microbial cultures with potential for applications in food production

Except for *Rhizopus* spp., the ability of food-grade microbial strains to detoxify mycotoxins is limited. Most toxicologically relevant mycotoxins are not detoxified by microbial species used in fermentation. Active strains have to be isolated from other sources. The first screening for mycotoxin-degrading microbes was carried out in the 1960s in the US Department of Agriculture, targeting aflatoxins (Ciegler et al. 1966). Many promising activities were reported but the results were rarely confirmed and advanced. Physical adsorption and enzymatic degradation were seldom differentiated. The unsatisfactory state of knowledge is reminiscent of the removal of aflatoxins by lactic acid bacteria. Numerous further bacterial and fungal species were identified in screening for the detoxification of aflatoxins but progress in elucidating mechanisms of their action was slow. It took 34 years to obtain indication that detoxification of aflatoxins by *Flavobacterium aurantiacum* is enzymatic (Smiley and Draughon 2000) yet the mechanism is still not known. New microorganisms removing mycotoxins from culture supernatant are continuously being described but follow-up studies are often missing. Degradation of AFB_1_ by *Rhodococcus erythropolis*, reported by Teniola et al. (2005) and Alberts et al. (2006) 10 years ago, is a typical example. It is to be hoped that the recent discovery of aflatoxin detoxification by *Rhizopus oryzae* and *Trichoderma reesei* (Hackbart et al. 2014) will not share this fate.

In some systems, studies of detoxification of mycotoxins at a molecular level revealed that the activities are not suitable for food processing. This applies in particular to the degradation of AFB_1_ by *Actinomycetes* sp. (Ciegler et al. 1966, Hormisch et al. 2004, Teniola et al. 2005, Alberts et al. 2006). The enzymes responsible for aflatoxin degradation in these bacteria are reductases depending on cofactor *F*_420_, which does not occur in microorganisms used in food processing (Taylor et al. 2010). Anaerobic de-epoxidation of trichothecenes by ruminal and intestinal microflora is another example. The activity is known since 1983 (Yoshizawa et al. 1983), the first active pure culture was obtained in 1997 (Binder et al. 1997) but the mechanism and enzyme(s) involved remain unknown.

Yeast used to protect fruits from fungal spoilage may enzymatically destroy the mycotoxin PAT, produced by *Penicillium* species in infected fruits. *Rhodosporidium paludigenum*, which is a yeast species studied for fruit protection, degrades PAT into less toxic desoxypatulinic acid (Zhu et al. 2015a). However, treatment of infected apples and pears with *R. paludigenum* increased the PAT content of the fruits, probably by triggering a stress response in PAT producers (Zhu et al. 2015b). Caution is therefore advised when biological control agents are applied to food commodities. Another example of potentially undesirable side effects of biological control provides ZEN. Because the mycotoxin protects its producer against mycoparasites and competitors (Utermark and Karlovsky 2007), applications of biological control agents to grain contaminated with ZEN-producing *Fusarium* species might induce increased ZEN production.

Laccases and peroxidases can degrade diverse organic compounds. Degradation products of aflatoxins by peroxidase from white-rot fungus *Phanerochaete sordida* were elucidated (Wang et al. 2011). In most cases, however, the mechanisms of detoxification remained unknown. For instance, Alberts et al. (2009) demonstrated degradation of AFB_1_ by laccases of several fungal species and showed that the products were not mutagenic but they have not determined their structures.

The list of microorganisms reported to detoxify mycotoxins is long; we refer to reviews on microbial degradation of mycotoxins in general (Karlovsky 1999; McCormick 2013; Hathout and Aly 2014) and on trichothecenes (He et al. 2010; Karlovsky 2011). New detoxification mechanisms for DON have been discovered recently (Ikunaga et al. 2011; Ito et al. 2013; He et al. 2015, 2016). Although the enzymes involved are still unknown, the use of these strains for the decontamination of food commodities appears promising. As holds for all microorganisms used in food processing, microbial strains for mycotoxin mitigation in food would require regulatory approval.

## Commodities

In this section, examples of raw materials and commodities are given that suffer from high mycotoxin contamination and for which mitigation strategies were studied.

### Cereals and derived products

Cereals are staple food worldwide. They are the primary source of carbohydrates and the main source of foodborne mycotoxin exposure. The degree of fungal penetration into the endosperm of grains is reflected in the redistribution of mycotoxins in milling fractions (see section “Physical processing methods”). Due to high mycotoxin concentrations in surface tissues of grains afflicted with Fusarium head Blight, sorting, cleaning, dehulling, and debranning reduce mycotoxin contamination of the flour. A large fraction of mycotoxins can be removed with damaged kernels, fine material, and dust (Cheli et al. 2013). The efficiency of such cleaning in mycotoxin reduction was demonstrated for T-2 and HT-2 toxins (Schwake-Anduschus et al. 2010), fumonisins (Saunders et al. 2001), and DON (Lancova et al. 2008a). Scudamore and Patel (2000) reported effect of cleaning on the content of aflatoxins and fumonisins. In the last decade, several authors reported DON reduction through debranning (e.g., Aureli and D’Egidio 2007). The concept was extended to masked mycotoxins such as DON-3-glucoside (Kostelanska et al. 2011). Recently, the fate of fumonisins along the entire corn meal production chain has been elucidated (Generotti et al. 2015b). Fumonisin content decreased by 40 % in cornmeal semolina; the reduction was less pronounced in corn flour and middlings.

Aflatoxins in cereal matrix can be reduced by soaking (with or without organic acids), cooking/heating, or steaming (Samarajeewa et al. 1990). Ordinary cooking of rice contaminated with AFB_1_ resulted in a reduction of 34 %; more than 70 % were destroyed by pressure cooking (Park and Kim 2006). The reduction of aflatoxin content by extrusion depends on the presence of additives, moisture level and the applied temperature/pressure; an efficiency of 50 to 80 % can be achieved. Similar effects were reported for OTA in bakery products (Scudamore et al. 2004).

Nixtamalization, which hydrolyzes ester bonds of fumonisins, reduced FB_1_ content of tortillas by 82 % (Dombrink-Kurtzman et al. 2000). Mycotoxins were retrieved mainly in the steeping and washing water.

Gamma and electron-beam irradiation was evaluated for the reduction of trichothecenes in grains (O’Neill et al. 1993). DON and 3-acetyl-DON were more efficiently destroyed in aqueous solution than on maize kernels.

The effect of bread baking on DON is controversial. While reduction by more than 50 % was observed in one study (Voss and Snook 2010), no changes in DON concentrations during baking was reported by others (Lancova et al. 2008a). The effect of the fermentation of dough on mycotoxins was also evaluated by Vidal et al. (2014). OTA remained stable, whereas DON concentration increased from unkneaded mix to fermented dough and decreased during baking. Zachariasova et al. (2012) observed the opposite: DON concentration decreased from flour to fermented dough and increased during baking. Other authors (Bergamini et al. 2010; Suman et al. 2012, 2014; Generotti et al. 2015a) confirmed the reduction of DON content during baking with the help of a Design of Experiments approach in a pilot plant and industrial production for bread, rusks, and crackers. Baking time and temperature were the key factors of DON reduction. Protease and xylanase used in the bakery industry released additional DON from the matrix during kneading and fermentation of dough (Simsek et al. 2012).

ZEN was reported to remain stable during bread baking (Cano-Sancho et al. 2013) but another study estimated ca. 40 % loss in bread and 20 % in biscuits (Alldrick and Hajšelová 2004). Numanoglu et al. (2013) constructed a kinetic model of ZEN degradation in maize bread during baking.

Nivalenol degradation accelerated with increasing bakery processing temperature (Bretz et al. 2005). Degradation of T-2 and HT-2 toxins by 20–30 % during bread baking was reported (Monaci et al. 2011) but no degradation was observed in another study (Schwake-Anduschus et al. 2010).

As shown earlier, studies of the fate of DON, ZEN, and T-2 during baking within the last 30 years left contradictory conclusions. Results on fumonisins are less extensive but equally contradictory. The first study, conducted more than 20 years ago, reported high losses of fumonisins in heated corn (Scott and Lawrence 1994). A more recent study (Numanoglu et al. 2010) found no significant reduction in the content of DON, ZEN, and fumonisins in traditionally produced Turkish maize bread. Finally, (Bryła et al. 2014) demonstrated reduction of fumonisins by 30 % during baking of gluten-free bread.

Studies of the fate of the depsipeptide mycotoxins enniatins (ENNs) and beauvericin (BEA) during processing of cereals are limited. Vaclavikova et al. (2013) showed that ENNs levels dropped during bread baking to 30 %. Meca et al. (2012) observed BEA degradation from 20 to 90 % in crispy breads during heat treatment and fermentation.

Ergot alkaloids in cereals attract increasing attention of food safety authorities (EFSA 2012). Reduction of sclerotia by up to 80 % can be achieved by winnowing previous to the milling, resulting in substantial reduction of ergot alkaloid content (Berg et al. 1995). Recent incidents of contamination with ergot alkaloids above tolerable levels were reported particularly in small (organic) enterprises (Masloff 2006). An explanation is that equipment for optical sorting is too expensive for small enterprises (Dusemund et al. 2006). Processing sclerotia-contaminated flour in bread, pancakes, or noodles result in a loss of the toxicologically relevant (R)-isomers. Complete decomposition of (R)-isomers of six predominant ergot alkaloids was observed during baking of whole wheat bread, whereas rye bread and triticale pancakes retained 85 and 74 % of these compounds, respectively (Scott and Lawrence 1982). A recent work on biscuits reported degradation and epimerization of up to 30 % ergot alkaloids to less toxic forms (Merkel et al. 2012).

In pasta, the solubility of mycotoxins in boiling water determines the level of consumer exposure. According to Visconti et al. (2004), most DON was extracted from pasta to cooking water. Brera et al. (2013) observed DON reduction in semolina in dry and cooked pasta by 8 and 41 %, respectively.

### Cocoa-chocolate

Cocoa is grown in West Africa, Asia, and Latin America mainly as raw material for chocolate production. Suboptimal storage and processing conditions in producing areas frequently cause fungal contaminations producing aflatoxins and OTA (Copetti et al. 2014). In order to minimize the OTA contamination of cocoa, cocoa-producing countries are developing new post-harvest treatment guidelines. OTA is concentrated in beans shells and therefore toxin levels in the nibs are low: mechanical shelling removed 48 % of OTA (Gilmour and Lindblom 2008), while shelling by hand reduced OTA to between 50 and 100 % (Amezqueta et al. 2005).

The first step of cocoa processing is opening the harvested pods at the farm site; then, the beans are fermented naturally by yeasts and bacteria. Experiments in Brazil (Copetti et al. 2012) demonstrated the importance of organic acids produced by fermentative bacteria in suppressing the growth of fungi with the potential to produce OTA. Fermented beans are dried in the sun on wooden platforms or on the ground. The OTA level significantly increases during transition from fermentation to drying (Dano et al. 2013). Drying must be therefore conducted as rapidly as possible. In processing plants, dried beans are broken and winnowed to obtain de-shelled kernels (nibs). The nib is sterilized with steam and roasted directly without (natural) or with addition of alkali to develop the final flavor and color. The temperature reaches 100 to 120 °C for a period of 15 to 70 min; it is not expected that OTA is degraded significantly in this step (Mounjouenpou et al. 2012). However, degradation of OTA by 17 to 40 % was reported in different experiments (Manda et al. 2009; Copetti et al. 2013).

The effect of roasting on aflatoxins in cocoa was evaluated by Mendez-Albores et al. (2013); roasting cocoa beans at 250 °C for 15 min reduced aflatoxin content by up to 71 %. Mycotoxin levels in alkalized cocoa powder tend to be reduced (Copetti et al. 2011; Turcotte et al. 2013). Alkalization appeared to be more effective in reducing aflatoxin than OTA. Results of Mendez-Albores et al. (2013) showed reduction in aflatoxin content in cocoa liquors due to the thermal-alkaline treatment up to 98 %. Turcotte et al. (2013) reported that OTA and AFB_1_ occurred ubiquitously in natural and alkalized cocoa, decreasing progressively from cocoa liquor to baking chocolate, to dark chocolate, and milk chocolate; no OTA was found in cocoa butter. Overall, it can be inferred that processing cocoa bean to chocolate leaves negligible concentrations of OTA and aflatoxins in the final product. In addition to the degradation described earlier, production of chocolate includes addition of other ingredients (e.g., milk products and sugars) which further dilutes mycotoxins in the final commodity.

The European Commission currently considers that introducing maximum limit of OTA in cocoa or cocoa products does not appear necessary for the protection of public health (European Commission (EC) Regulation No. 1881/2006). Indeed, samples of cocoa containing more than 2 ng/g of these mycotoxins can rarely be found.

### Coffee

Green coffee beans are one of numerous food commodities significantly contaminated with OTA (Speijers and van Egmond 1993). Coffee and cocoa beans are hygroscropic and thus vulnerable to contamination with OTA during storage and transport (Magan and Aldred 2005). Scudamore (2005) reviewed the effect of roasting and brewing of coffee on the level of OTA as compared to green coffee beans. OTA levels were drastically reduced during production of soluble coffee. The roast and the ground of coffee contained only 16 % of the concentration found in raw green coffee beans (Blanc et al. 1998). Similarly, Milanez (1996) reported 84 % of OTA reduction in processed beans. However, other investigators found lower OTA reduction (Leoni et al. 2000).

### Fruit juices

The major mycotoxin in fruit juices is PAT. The starting point of reducing PAT in apple-derived products is the selection of intact apples and the removal of rotten apples. Several studies have shown that PAT is stable in slightly acidic apple and grape juices but is decomposed during the production of cider (Moss and Long 2002). Alcoholic fermentation converts PAT into ascladiol, which is less toxic than PAT (Suzuki et al. 1971). A number of studies on the effect of different physical, chemical, or microbiological food processes on PAT concentrations have been performed (Leggett et al. 2001; Castoria et al. 2011; Zhu et al. 2015b). PAT reacts with sulfhydryl (thiol) groups of proteins, polypeptides, and amino acids available in certain food commodities such as cereals to form intra- and intermolecular protein cross links. These PAT adducts are not detected by conventional methods for PAT analysis (Fliege and Metzler 1999).

### Milk and other dairy products

AFB_1_ contaminating dairy feed may be metabolized in the animal into its monohydroxy derivative form aflatoxin M_1_ which is carried over into milk (Holzapfel et al. 1966). AFM_1_ or its metabolites can then contaminate subsequent dairy products. Distribution and stability of AFM_1_ during processing, ripening, and storage of Telemes cheese was studied by Govaris et al. (2001). Concentration of AFM_1_ in the curds was about four times higher than in milk but it fell in the cheese during ripening. In certain cheese kinds in Turkey, the concentration of AFM_1_ was, however, higher in the cheese than in bulk milk (Bakiri 2001). Fermentation of milk to yogurt at pH 4.6 and 4.0 reduced AFM_1_ concentration by 13 and 22 %, respectively; total loss of AFM_1_ after storage was 16 and 34 %, respectively (Govaris et al. 2002). In opposition with these findings, Yousef and Marth (1989) reported that AFM_1_ in fermented milk remained stable. Separation of milk components partitioned the toxin in accordance with its affinity for casein and the lack of solubility in fats.

### Vegetable oils

Mahoney and Molyneux (2010) assumed that aflatoxins are not found in vegetable oils but there is increasing evidence that this does not apply to non-purified or crude vegetable oils (Shephard et al. 2011). High incidences of aflatoxin contaminations in edible vegetable oils were even reported (Bordin et al. 2014). The different processes used for vegetable oil extraction may partially explain these discrepancies. Edible vegetable oils indeed can be extracted from oleaginous material either by mechanical pressing or by solvents.

Distribution of mycotoxins from steeped corn to corn germs in wet milling depends on their solubility. Water-soluble mycotoxins such as DON were found at high concentrations in steep liquor but at low levels in the solid (germ, fiber, and gluten) fractions. The inverse is true for ZEN, which is relatively insoluble in water (Table 2). ZEN may occur in maize germ oil but investigations about the effect of processing of oils on ZEN are limited, except for UV light exposure during storage (see section “Irradiation”). Abalaka and Elegbede (1982) reported that only 10–20 % of aflatoxins were transferred from groundnuts and cotton seeds to crude oil. Similarly, Parker and Melnick (1966) found most aflatoxins after extraction of oil from peanut and maize in defatted meal. In their work, corn oil extracted with chloroform contained more aflatoxins than oil extracted with hexane. Refining based on alkaline neutralization, washing, bleaching, and deodorization was reported to remove mycotoxins from oil (Kamimura et al. 1986). There are no studies in the literature relating degumming to the presence of aflatoxins.

In the deacidification step of refining, fatty acids are removed using an alkali (chemical refining) or water vapor (physical refining). Alkaline treatment is effective in degrading aflatoxins (see section “Treatment with bases”). Kamimura et al. (1986) evaluated the efficiency of the processing steps of refining vegetable oils contaminated with mycotoxins and observed that after treatment with sodium hydroxide, AFB_1_ and AFB_2_ were significantly reduced, DON and NIV were present only in traces and AFG_1_ and AFG_2_ were completely eliminated. These results confirmed an earlier finding by Parker and Melnick (1966) that sodium hydroxide efficiently removed aflatoxins from peanut oil. ZEN is removed below a level of concern from corn germ oil by alkaline treatment at pH 9–10 (Slope et al. 2013).

Bleaching consists of introducing an adsorptive bleaching material, called bleaching earth or clay, under vacuum condition and high temperature, and subsequent removal of the agent by filtration. Parker and Melnick (1966) found that bleaching reduced aflatoxin levels in peanut and corn oils below 1 μg/L. Kamimura et al. (1986) reported that bleaching of vegetable oil eliminated trichothecenes and aflatoxins but not ZEN.

The last step of the refining process of vegetable oil is the deodorization step, which is a codistillation process using water vapor under high temperature (220–270 °C) and low pressure (0.1–0.7 kPa). Conditions adopted in the deodorizing process can lead to complete removal of aflatoxins from vegetable oils, as Kamimura et al. (1986) showed for artificially contaminated vegetable oils. The levels of trichothecene and ZEN were reduced, too. Thus, it is possible to ensure safe edible vegetable oil provided it is properly processed.

### Ethanol and beer

In process where fermentation is followed by distillation, mycotoxins are not present in the alcohol fraction but may be increased in the spent grain product. AFB_1_ was decreased by 47 % after cooking and fermenting contaminated corn or wheat (Dam et al. 1977), although its concentration in the solids after distillation was higher than in the starting grain. No ZEN from contaminated corn appeared in ethanol but ZEN concentration in the solids after fermentation doubled. Two lots of corn contaminated with 15 and 36 mg/kg of FB_1_ were fermented for ethanol production (Bothast et al. 1992). Analysis of the various fermentation products showed that there was little fumonisin degradation during fermentation. No FB_1_ was found in distilled ethanol or centrifuged solids. Most of the fumonisins were distributed over distillers’ dried grains, thin stillage, and distillers’ solubles.

Most mycotoxins can survive brewing and end up in the beer; DON is found at highest concentrations (Scott 1996). Lancova et al. (2008b), however, reported that even larger amounts of DON in beer were present in the form of DON-3-glucoside. Schwartz et al. (1995) found that in the brewing process both ZEN and 15-acetyl-DON increased during germination of barley. Eighty to 93 % of DON from malt grist was found in the beer; 60 % of ZEN and 18 % of 15-acetyl-DON were found in the spent grains. Scott and Lawrence (1995) examined the losses of OTA as well as of FB_1_ and FB_2_ in fermentation of wort. Up to 21 % of OTA but negligible amounts of fumonisins were taken up by the yeast, indicating that OTA and fumonisins could contaminate beer. Fumonisins were later indeed detected in domestic and imported beer in Canada (Scott et al. 1993). Most recently, the fate of 14 mycotoxins during beer brewing was investigated by Inoue et al. (2013). After brewing, the levels of aflatoxins, OTA, FB_2_, PAT, and ZEN dropped below 20 %. ZEN and PAT were apparently metabolized to less toxic compounds. Trichothecenes survived brewing at more than 50 % of their initial concentration. Vaclavikova et al. (2013) reported that enniatins from contaminated wheat and barley were not transferred into beer; these hydrophobic mycotoxins remained adsorbed on spent grain.

### Dried fruits, nuts, and spices

Food safety is significantly affected by mycotoxin contamination of dried fruits, nuts, and spices. Dry fruits such as raisins, sultanas, figs, apricots, and dates are consumed worldwide. Cultivation and processing of these fruits in warm climates rise mycotoxin risk, especially concerning aflatoxins and ochratoxin A. The pH of fruits ranging from 2.5 to 5.0 is the most important factor affecting spoilage of fruits by microorganisms. Fruits become increasingly susceptible to fungal invasion during ripening, as the pH of the tissue increases and skin layers soften (Drusch and Ragab 2003). Other factors contamination level are harvesting and drying conditions and moisture content (Bullerman et al. 1984). Physical cleaning and separation, where the mold-damaged kernel/seed/nuts are removed, can result in 40–80 % reduction in aflatoxin level. Dry and wet milling may redistribute aflatoxins into less utilized fractions.

In groundnuts, higher levels of aflatoxin are associated with small, immature pods. Removing these pods reduces aflatoxin concentration in shelled lots (Dorner 2008). To remove foreign material and unshelled pods, shelled peanuts are subjected to gravity separation. Because highly contaminated kernels are less dense, this process reduces aflatoxin contamination (Davidson et al. 1981). Shelled kernels can be further separated by size through a series of slotted screens; generally, aflatoxins are associated with smaller-sized kernels (Whitaker et al. 2005). Further, aflatoxin reduction can be obtained by blanching combined with electronic color sorting (Cole et al. 1995) and sorting with the help of IR and UV spectroscopy (Durmus and Kalkan 2016). Blanching and color sorting is used for other nuts such as pistachios, too.

Among dried fruits, figs are the most challenging. Removal of damaged fruits, solar drying, fluorescence sorting, and treatment with sulfur dioxide are effective mycotoxin mitigation strategies (Scott and Trucksess 2009). Sun-drying figs are often practiced in tropical countries but because it is slow, it allows proliferation of molds producing mycotoxins. Ozay et al. (1995) showed that dipping figs in solution of metabisulfite or sorbate or in hot water followed by dehydration reduced fungal colonization and aflatoxin content. Comparison of different drying systems with sun-drying revealed that ultrasound treatment combined with osmotic solutions is most effective (Villalobos et al. 2016).

Very recently, the use of cold atmospheric plasma to destroy aflatoxins in dehulled hazelnuts was reported, with over 70 % aflatoxin reduction achieved by 12 min of treatment (Siciliano et al. 2016). Another recent work demonstrated that roasting pistachio nuts with lemon juice and/or citric acid destroyed over 90 % AFB1 (Rastegar et al. 2016).

To the best of our knowledge, the effect of food processing on mycotoxin levels in spices was not studied. A moderate reduction of OTA and aflatoxin content in pepper by gamma radiation was reported (Jalili et al. 2012).

## Implications and outlook

Mycotoxin contamination of food commodities, especially of staple foods, poses a serious threat to human health. Efficient reduction of mycotoxin exposure via food products requires the utilization of all available technologies from good agricultural and storages practices and selection of raw materials suitable for human consumption to the application of food processing technologies including biotechnology. Today’s consumers are keenly aware of the importance of food for their health. Their perception of food safety has been heavily biased towards man-made pollutants but toxic compounds of natural origin are slowly gaining attention. Food industry has recognized the trend, as intellectual property protection efforts in mycotoxin detoxification show (He and Zhou 2010).

Mitigation of mycotoxins as a side effect of established food processing techniques, such as fermentation of apple juice or nixtamalization of maize flour, should be utilized whenever possible. Development of new techniques dedicated to mycotoxin mitigation will, however, require extensive research. The impact of mycotoxin mitigation processes on the nutritional composition and organoleptic quality of food and their influence on other contaminants such as acrylamide (Anese et al. 2009) have to be assessed. The advantage of reducing mycotoxin levels has to be weighed against the loss of material and/or nutrients. Novel physical and chemical treatments (cold plasma) and novel detoxification agents (microbes or purified enzymes) for mycotoxin mitigation in food would have to undergo regulatory approval, which implies a risk analysis. European regulation 1881/2006 provides direction in what is likely to be acceptable; similar regulations operate outside Europe (refer also to EC regulation 2015/786).

Disappearance of a parent mycotoxin does not necessarily mean detoxification, if the toxin is converted into a form that escapes detection, yet remains toxic. Often, the mechanism of mycotoxin transformation is not fully understood, the products have not been characterized, and their bioavailability and toxicity compared to the parent compound has not been assessed. Limited toxicological investigations on mycotoxin degradation products were restricted to in vitro and acute in vivo studies which provide insufficient information regarding the safety at chronic low level exposure. New predictive tools in toxicology (Schilter et al. 2014; Kavlock et al. 2012; Cozzini and Dellafiora 2012) may be helpful in identifying transformation or degradation products requiring detailed toxicological investigation. Without such knowledge and as a precautionary approach, risk assessment has to assume that all mycotoxin forms have the same bioavailability and toxicity as the respective parent compound (EFSA 2014).

While detoxification of mycotoxins has been studied extensively, little is known about the potential of food processing to increase or hide mycotoxin exposure. Chemical and physical treatments applied to food may release mycotoxins from masked forms and make them bioavailable or convert mycotoxins into forms not detectable by conventional analytical methods (Rychlik et al. 2014) while retaining their toxic potential (Suman and Generotti 2015) or stimulate contaminating fungi to mycotoxin production, e.g., during steeping of barley. Analytical tools for mycotoxins transformed by processing (structural modification, binding to food matrix) need to be developed.

Most research on mycotoxins focused on regulated mycotoxins. The recent unexpected discovery of the fungus *Stachybotrys chartarum* in culinary herbs (Biermaier et al. 2015) showed that the scope has to be broadened: *S. chartarum*, so far known mainly from water-damaged walls, produces macrocyclic trichothecenes of higher acute toxicity than any regulated mycotoxin. Highly toxic metabolites of the fungus *Stenocarpella maydis* recently discovered in maize grains provide another example of toxicologically relevant non-regulated mycotoxins in food (Rogers et al. 2014). Even well-studied fungal metabolites may raise new food safety concerns, such as AAL toxin of *Alternaria alternata* if it occurs in tomato juice (Karlovsky 2016). Genome sequencing revealed that fungi-contaminating food commodities have the potential to produce 30–60 secondary metabolites each, some of which might turn out to be mycotoxins. Mitigation strategies have to be adapted to newly discovered mycotoxins, once their toxicity and level of exposure have been assessed. Undoubtedly, the list of regulated mycotoxins will grow.

The large number of combinations of processing/commodities/mycotoxins calls for prioritization among applications on which to focus further research efforts. Criteria for prioritization should be consumption of the contaminated commodity (staple foods and commodities consumed by sensitive population groups like young children), occurrence at high levels in such commodities and unfavorable toxicological profiles. Different geographical regions and target groups require different prioritizations. An example is fumonisin contamination of maize: in Africa, maize is a staple food, causing human exposure to fumonisins that would exceed the tolerable daily intake (TDI) even if EU limits of contamination could be achieved (Shephard et al. 2007; JECFA 2012). Celiac disease patients in industrialized countries consume above-average amounts of maize; it was shown that their dietary exposure to fumonisins clearly exceeds average levels (Dall’Asta et al. 2012). Most consumers in Europe, however, would not exceed TDI for fumonisins even if they consume maize exceeding the EU limit by an order of magnitude. Guidelines have been elaborated on how to identify relevant targets for mitigation, determine the effectiveness of mitigation measures, and assess the risk of unintended consequences (van der Fels-Klerx et al. 2014).

## Conclusions

Food processing can reduce mycotoxin exposure by destroying or eliminating mycotoxins, by transforming them into less toxic derivatives, by adsorbing mycotoxins to solid surfaces or by reducing their bioavailability due to chemical attachment to food matrix structures. Complete elimination of mycotoxins from food product by processing can rarely be achieved.

Several processing techniques of proven value (mostly physical treatments) have been in use for a long time. These are the only mycotoxin mitigation methods currently applicable to human food. Few chemical and biotechnological techniques reducing mycotoxin content have been approved for animal feed but many promising strategies remain at an experimental stage. In addition to mycotoxin derivatives modified by microorganisms or plants (EFSA 2014), the risk assessment of mycotoxins in food has to include mycotoxin forms resulting from food processing. Before a novel mycotoxin decontamination technique is approved, chemical identity and toxicity of the reaction products have to be determined. Availability of analytical methods which permit reliable detection of these products is a key prerequisite for risk assessment. Bioavailability and toxicity of transformation products should be assessed using a systematic approach and generally acknowledged testing systems. This in turn allows prioritizing those which require more detailed toxicological assessment and the choice of specific, adequate risk assessment options. Recently established legislative criteria for detoxification processes applied to feed may serve as a model (EC 2015). In the absence of adequate toxicological data, mycotoxin forms generated during processing must be assumed to have the same toxicity, bioavailability and carcinogenic potency as the respective parent compounds. The development of mitigation strategies should prioritize mycotoxins that regularly occur at high levels in highly consumed commodities and have unfavorable toxicological profiles. The ultimate goal of mycotoxin mitigation is to prevent adverse health effects caused by foodborne exposure to mycotoxins while preserving nutritional and organoleptic quality of food.
